# UV-C Light-Based Surface Disinfection: Analysis of Its Virucidal Efficacy Using a Bacteriophage Model

**DOI:** 10.3390/ijerph19063246

**Published:** 2022-03-10

**Authors:** Stefan A. Rudhart, Frank Günther, Laura Dapper, Boris A. Stuck, Stephan Hoch

**Affiliations:** 1 Department of Otolaryngology, Head and Neck Surgery, University Hospital Marburg, Philipps-University Marburg, 35043 Marburg, Germany; StefanAlexander.Rudhart@uk-gm.de (S.A.R.); boris.stuck@uk-gm.de (B.A.S.); 2 Department of Medical Microbiology and Hygiene, University Hospital Marburg, Philipps-University Marburg, 35043 Marburg, Germany; kristina.vigneri@uk-gm.de (F.G.); lauraisabel.dapper@uk-gm.de (L.D.)

**Keywords:** endoscopes, otorhinolaryngology, COVID-19, MS-2, UV light, D25, disinfection, surface disinfection, virus, adenovirus

## Abstract

Background: The reprocessing of medical devices has become more complex due to increasing hygiene requirements. Previous studies showed satisfactory bactericidal disinfection effects of UV-C light in rigid and flexible endoscopes. Especially in the context of the current COVID-19 pandemic, virucidal properties are of high importance. In the present study, the virucidal efficacy of UV-C light surface disinfection was analyzed. Methods: MS-2 bacteriophages were applied to the test samples and irradiated by UV-C light using the UV Smart D25 device; unirradiated test samples were used as controls. A dilution series of the samples was mixed with $1\;\times \;10^{8}\;$*Escherichia coli* and assayed. Results: 8.6 × 10^12^ pfu could be harvested from the unprocessed test samples. In the control group without UV-C exposure, a remaining contamination of 1.2 × 10^12^ pfu was detected, resulting in a procedural baseline reduction rate with a LOG_10_ reduction factor of 0.72. The LOG_10_ reduction factor was found to be 3.0 after 25 s of UV-C light exposure. After 50 and 75 s of UV-C radiation LOG_10_ reduction factors 4.2 and 5.9, respectively, were found, with all reductions being statistically significantly different to baseline. Conclusions: The tested UV system seems to provide a significant virucidal effect after a relatively short irradiation time.

## 1. Introduction

**Figure 1:**
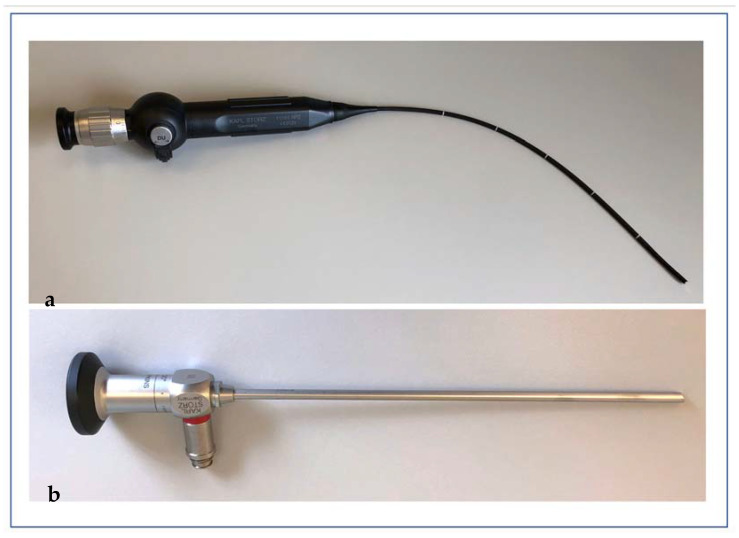
Flexible (**a**) and rigid (**b**) otorhinolaryngological endoscope for examination of the upper aerodigestive tract.

Throughout the medical sector, a wide variety of medical devices have to be subjected to high-level disinfection before reuse. These products are so-called semi-critical medical devices, as defined by the Spaulding classification [1]. Semi-critical medical devices enter the orifices of a patient’s body without penetrating the patient’s skin barrier. Accordingly, high levels of bacterial and viral contamination on these surfaces as well as a higher risk of cross infections must be expected compared to non-critical medical devices that are only used on the patient’s skin (e.g., stethoscopes). In otorhinolaryngology (ORL), there is the potential for several viruses to be transmitted through endoscopes, including carcinogenic viruses such as the human papilloma virus (HPV), which plays an important role in the development of head and neck cancer, which has seen increasing incidence in recent years [2]. In general, several viruses, such as coronaviruses, first invade the mucosa of the upper aerodigestive tract and multiply there and are further spread by droplet infection [3,4,5]. Thus, it is understandable that the use of medical devices that have specific usage in the upper aerodigestive tract pose a particular risk of cross infection with potentially highly pathogenically and carcinogenic viruses to patients. In particular, these devices include rigid and flexible endoscopes (Figure 1), which are frequently used in ORL for the examination of the aforementioned area. According to the previously mentioned Spaulding classification, these endoscopes are classified as semi-critical medical devices. Due to their frequent use, it is obvious that reliable, cost-effective, and easy-to-use reprocessing methods for endoscopes in ORL are currently of great importance. A large number of techniques are used for endoscope disinfection, but no international standard has been established thus far. Well-established, high-level methods are often either expensive and/or time consuming and are often also user-dependent, which may result in these methods being unreliable and challenging for everyday clinical use, particularly in an ORL outpatient clinic with a high patient load.

**Figure 2:**
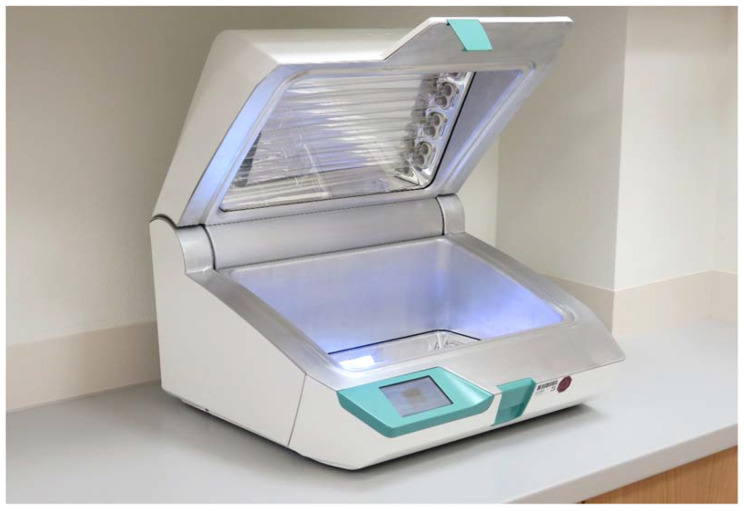
D25 UV light system for endoscope disinfection.

UV light has been used for surface disinfection and patient treatment for more than 120 years. One of the first users was Nils Ryberg Finsen, a Danish physician, who was awarded the Nobel Prize for his work a few years later [6,7]. After 1945, UV lamps were used in continuous mode to prevent the outbreak of pathogens in medical facilities [8]. Today, UV light is commonly used to disinfect drinking water, and compared to chemical methods, it has the benefits of being nontoxic and does not impart a taste or aroma to the water [9]. Recent studies have revealed very good effects of UV light in disinfecting medical surfaces. Its effectiveness against problematic multi-resistant germs or biofilm-forming bacteria should be emphasized in this context [10,11]. Further satisfactory results were achieved for viruses with a germ load reduction of at least LOG_10_ 3 in the surface disinfection context, which was dependent on the energy applied per area and the wavelength [12,13]. In a previous study, a UV light system (UV Smart Technologies B.V., Rijswijk, The Netherlands) for the reprocessing of rigid endoscopes was tested with regard to its bactericidal properties, showing promising results (Figure 2) [14]. In that study, an absolute germ reduction in standardized test specimens of about LOG_10_ 7 was found. Moreover, after use on patients, almost all of the endoscopes were sterile and nearly protein-free after reprocessing. According to the manufacturer, the arrangement of UV lamps in the D25 UV light system leads to high energy output per area, which results in good virucidal properties. In this context, the present study was designed to test and analyze the actual virucidal effect of the D25 UV light system through the use of MS-2 phages. MS-2 phages are commonly used as a surrogate for stable viruses, e.g., adenoviruses, which are considered to be one of the most difficult viruses to eradicate [15,16].

## 2. Materials and Methods

### 2.1. The D25 UV Light System

The D25 UV light system (UV Smart Technologies B.V., Rijswijk, The Netherlands) disinfects medical devices using UV-C light. It operates at a wavelength of 253.7 nm. During the disinfection cycle of 25 s, the D25 delivers a dose of 6.872 mW/cm^2^ per second. A length of 25 s per disinfection cycle is preset by the manufacturer, but this can be individually adjusted to reach higher energy levels per cycle if required. The applied UV-C radiation causes DNA or RNA destruction in irradiated microorganisms. UV light is not able to penetrate solid substances, meaning that surfaces that should be reprocessed need to be visually clean before using the D25. Therefore, a water-based precleaning is recommended by the manufacturer when disinfecting devices with smooth surfaces. No further chemicals or liquids are needed for reprocessing.

For operator and patient safety, the UV-C light cannot escape the UV system because of its box-based design, and the UV lamps turn off if the lid is opened during the disinfection process. Devices with a maximum size of 15 cm × 22.5 cm × 38.0 cm are suitable for the disinfection chamber, and this limit should not be exceeded. A total of 8 UV light-applying lamps are installed on both the top and bottom of the system and are protected by a Borosilicate glass panel. The distance from the lamps to the object is 150 mm. The sides of the chamber are lined with special patented reflective metal elements (Impelux™ Technology, UV Smart Technologies B.V., Rijswijk, The Netherlands) to ensure the most efficient UV light distribution.

### 2.2. Statistics and Ethics Approval

Statistical analysis and graph preparation was performed using ANOVA according to the Kruskal–Wallis method using SPSS and Excel 2019 software (IBM, Armonk, NY, USA; Microsoft Corporation, Redmond, WA, USA). A *p*-value of less than 0.05 was considered to be statistically significant. The *p*-value refers to a comparison between the unirradiated control samples and the irradiated samples. The ethics committee of the Medical Faculty of the Philipps-Universität Marburg was notified of this trial. According to their statement, a formal approval was not necessary as no patients or animals were included [file number: ek_mr_6_12_19_stuck].

### 2.3. Virucidal Testing

The effectivity of the UV-C irradiation was tested in six independent experiments, except the disinfection cycle with 75 s with only three test series. Each sample was tested in triplicate using glass slides and polystyrene dishes. A phage suspension of bacteriophage MS2, NCTC 12487 as a surrogate for stable viruses with high tenacity was prepared at 12 × 10^10^ plaque forming units (pfu)/mL in peptone saline solution (Merck, Darmstadt, Germany).

As harvesting the phages from the test samples is associated with a dilution of the suspension applied to the test bodies, the phage concentration in the initial phage suspension cannot be used as a baseline concentration. Therefore, 100 μL of a standardized phage suspension was spotted in thin layers on the respective surface of 6 test bodies and immediately rinsed off, using 1 mL of peptone saline solution. The phage concentration detected in these non-irradiated and unprocessed test samples was used as a baseline value for the irradiated samples and the control sample. In a second step, six test bodies prepared with phages were then dried over the course of 30 min at room temperature and rinsed off with peptone saline solution and served as a control sample. In a third step, 16 samples were prepared with phages, dried as described above and additionally irradiated for up to three cycles (each 25 s) using the UV Smart D25 device.

After the phages had been rinsed off using 1 mL peptone saline solution, dilution series were prepared starting from 1:10^1^ to 1:10^13^ for each sample. In the next step, 1 mL of each dilution was mixed with 100 μL of a peptone saline solution containing 1 × 10^8^ *Escherichia coli* K-12 Hfr NCTC 12486, and it was plated out on TYGA plates tryptone (Sigma Aldrich, Taufkirchen, Germany), yeast extract (Oxoid, Wesel, Germany), NaCl (Carl Roth, Karlsruhe, Germany), and agar (VWR, Darmstadt, Germany). In parallel, confirmation samples containing RNase solution (Quiagen, Hilden, Germany) were prepared using the same procedure but with the additional step of adding 100 µL RNase solution (1 mg/mL) before plating. After 24 h incubation at 37 °C, the plaque formation in *E. coli* were counted, and the phage concentration was determined using the formula

Cpfu = ((N − N.RNase) · F)/n.

N—total number of counted plaques on WG49 plates. 

N.RNase—the total number of plaques counted on WG49 plates with RNase.

*n*—number of parallel determinations.

F—dilution factor.

The LRF_10_ was calculated as a surrogate for the virucidal effectivity of the device for the irradiated samples using the formula:

LRF_10_= LOG_10_ (Cpfu baseline/Cpfu irradiated samples)

Since the non-irradiated control samples also showed a reduction in the bacteriophage titer due to unspecific processing (drying), the corresponding LRF_10_ for the control group (w/0) was calculated using the following formula:

LRF_10_ (w/0) = LOG_10_ (Cpfu baseline/Cpfu control samples)

**Figure 3:**
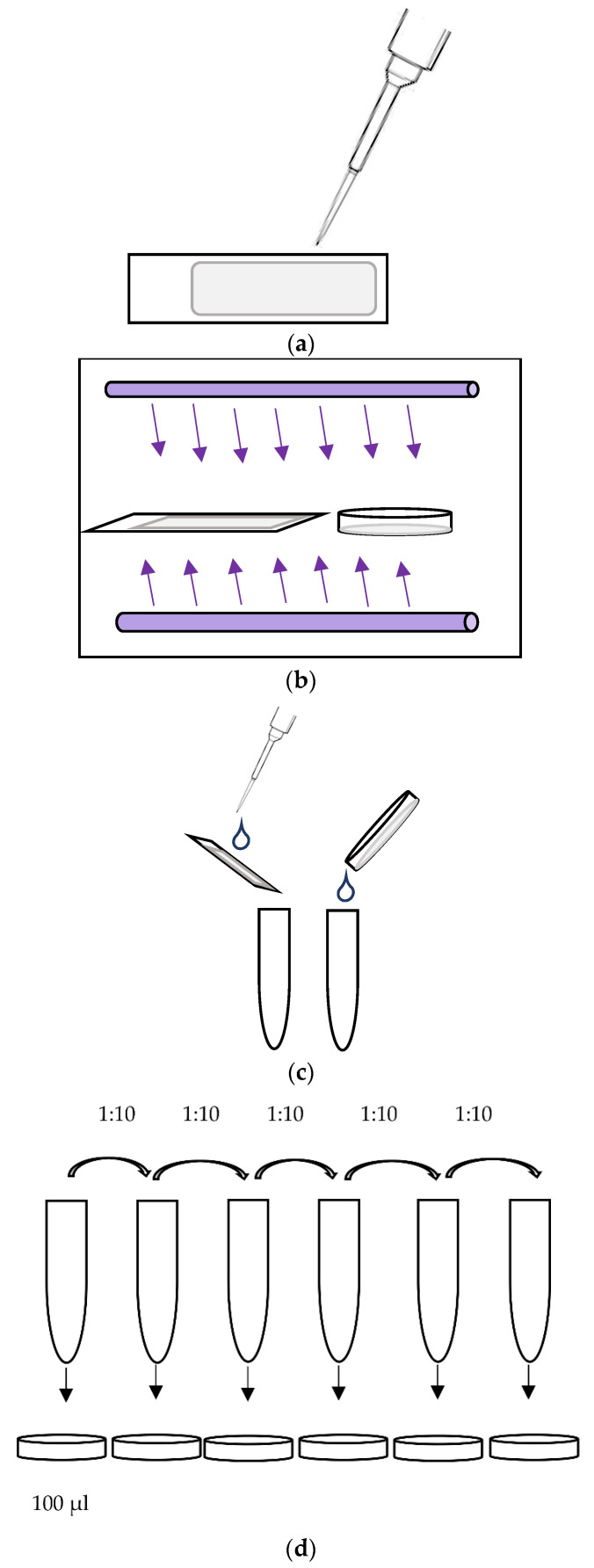

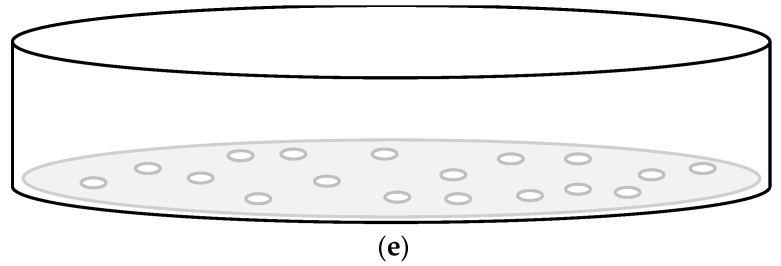
Schematic illustration of the virucidal testing workflow. (**a**) Application of phage suspension (100 µL) on cover slides. (**b**) Drying at room temperature and subsequent UV-C irradiation. (**c**) Rinsing off the surface off the test bodies using 1 mL of peptone saline solution. (**d**) Using dilution series and mixing 1 mL of each dilution with 100 μL peptone saline solution containing 1 × 10^8^ *Escherichia coli*. Plating the suspension (incubation at 37 °C for 24 h). (**e**) Plaque counting.

For a graphic illustration of the virucidal testing, please see Figure 3.

## 3. Results

The baseline concentration of the MS-2 bacteriophages was 8.6 × 10^12^ pfu (±1.7 × 10^13^, min 8.2 × 10^9^, max 4.3 × 10^13^) and 1.2 × 10^12^ pfu (±2.7 × 10^12^, min 4.2 × 10^9^, max 6.7 × 10^12^) in the control samples. Therefore, a mean LRF_10_ (w/0) of 0.72 (±0.54, min 0, max 1.38) served as a procedural reduction rate and was compared to the UV irradiated samples to assess the specific effects of UV-C exposure.

**Figure 4:**
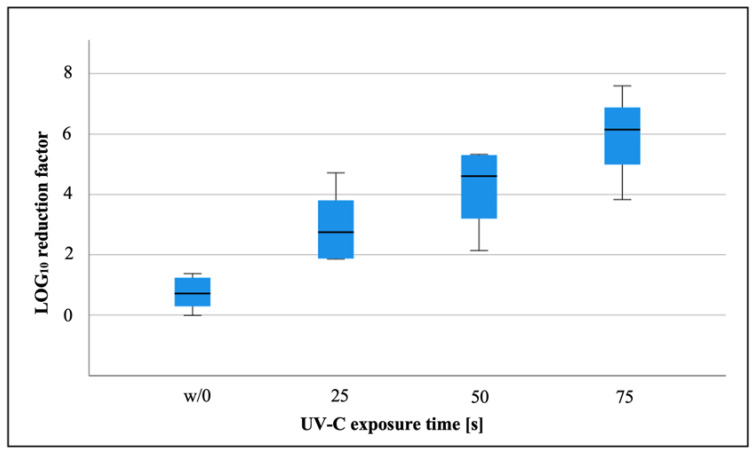
LOG_10_ reduction factor (LRF_10_) of the MS-2 bacteriophages in relation to the UV-C light exposure time in seconds (showing median values, interquartile range, minimum, and maximum). w/0= unirradiated control sample.

After 25 s of UV-C exposure, the mean bacteriophage titer was 3.1 × 10^9^ (±3.3 × 10^9^, min 1.2 × 10^6^, max 6.7 × 10^9^), which corresponded to a mean LRF_10_ of 3.0 (±1.2, min 1.9, max 4.7) compared to the unirradiated test samples. The mean MS-2 bacteriophage titer after 50 s of UV-C irradiation was 2.0 × 10^8^ (±2.9 × 10^8^, min 4.0 × 10^5^, max 7.4 × 10^8^), resulting in a mean LRF_10_ of 4.2 (±1.3, min 2.1, max 5.3). After 75 s of UV-C irradiation, a mean MS-2 bacteriophage titer of 2.6 × 10^6^ (±2.5 × 10^6^, min 1.1 × 10^6^, max 5.5 × 10^6^) was found, while the mean LRF_10_ was 5.9 (±2.0, min 3.8, max 7.6) (Figure 4). The difference in the LRF_10_ after 25 s of additional UV-C exposure was statistically significant (*p* = 0.041). 

Compared to the unirradiated control samples, a significant LOG_10_ reduction was found after 25 (*p* = 0.041), 50 (*p* = 0.01) and 75 s of UV-C irradiation (*p* < 0.01).

## 4. Discussion

In the context of the current COVID-19 pandemic, safe surface disinfection methods are highly relevant. Since a large number of human pathogenic viruses replicate in the upper aerodigestive tract, the relevance of reliable ORL-endoscope disinfection methods is even higher [3,4]. Many of the endoscope reprocessing methods that are currently used are highly user-dependent and are, thus, vulnerable to errors [17]. Furthermore, chemical-based disinfection methods pose a potential risk for the user. A potentially effective, non-chemical, and user-independent technique could be disinfection by means of UV-C radiation. UV-C radiation causes the dimerization of thymine in DNA and uracil in RNA in the irradiated microorganisms as well as RNA-protein cross-links, which leads to an inactivation [18,19]. In previous studies, the device tested here showed satisfactory results in terms of bactericidal efficacy for ORL-endoscopes [14,20]. The tested UV system was developed for the disinfection of rigid ORL-endoscopes (Figure 2). However, the reprocessing of ORL-endoscopes by UV light with a focus on virucidal aspects has not been investigated to date.

Previous studies have proven the virucidal efficacy of UV light against various viruses such as SARS-CoV-2 or influenza viruses [21,22]. Nevertheless, 9 min of irradiation time were needed for the sufficient elimination of the SARS-CoV-2 virus when using a titer of 5 × 10^6^ TCID 50/mL [21]. The required duration of action to reach a sufficient disinfection result seems to be related to the energy output of the applied UV radiation, the wavelength, and the initial virus titer. As well as spores, viruses seem to be the pathogens that are the most resistant to UV radiation. In the present study, an MS-2 bacteriophage model was used to simulate viral contamination with a very stable viral strain [15,23]. MS-2 phages are commonly used as surrogates for stable viruses, e.g., adenoviruses, due to their similar size, shape, and genome composition [15,16]. It should be noted that the results presented here show a high standard deviation in phage concentration. The high standard deviation is due to both the method variability and the biovariability caused by the instability of the phages. A high titer of bacteriophages of 8.6 ×10^12^ pfu was used to evaluate the absolute efficiency of the UV system investigated here. According to the manufacturer, the UV system itself is able to apply a light dose of 6.87 mJ/cm^2^ per second. In most studies, an almost linear increase in the bacteriophage LOG_10_ reduction with an increase in the applied light dose and therefore the time of irradiation is proven [15,24]. In the present study, a similar result could be observed, resulting in a higher LOG_10_ reduction by applying a higher light dose. Although a significant reduction in phage activity was detected after 25 s of UV disinfection, a LOG10 reduction of 4, which meets the requirements of the German regulatory authorities for the virucidal activity of disinfectants, was only achieved after 75 s of irradiation [25]. At this point, however, it should be mentioned that an LRF10 (w/0) of 0.72 was reached by the drying process alone. This must be taken into account when interpreting the results from a clinical perspective. Although the specific effects of UV-C radiation can only be assessed in comparison to the unirradiated control group, the unspecific procedural effects due to the drying process increase the effects of the entire process when using UV-C light in the clinical context. In our opinion, the main reasons for the effectiveness of the system tested here are the wavelength used, the short distance to the irradiated object, and the good light distribution in the system. In particular, the wavelength of 253.7 nm used appears to be the most effective wavelength in terms of eliminating both viruses and spores, which are the most difficult to eliminate by UV light [24]. Other wavelengths often showed poorer results for either spores or MS-2 phages as a surrogate marker for viruses [24]. Due to the short distance to the irradiated object and the wavelength used, a UV dose of 6.872 mW/cm^2^ could be applied per second.

In addition to the high virucidal efficacy shown here, the bactericidal efficacy of the D25 UV system has already been demonstrated in a previous study [14]. In this previous study, an absolute bactericidal efficacy of LOG_10_ 7 was found when positioning the test specimens according to the manufacturer’s specifications. In addition, in clinical testing, almost all of the endoscopes were germ-free and protein-free after precleaning and UV reprocessing. Furthermore, it is known that UV light-based reprocessing methods are effective against multi-resistant and biofilm-forming bacteria irrespective of the Gram stain or shape [8,26,27,28]. As well as its good disinfection properties, there are also some limitations for UV disinfection: Due to its physical mode of action, UV radiation does not penetrate solid masses or opaque liquids. Within ORL, upper aerodigestive tract secretions might be problematic. Therefore, in clinical use, a precleaning using a water-based tissue before UV disinfection is of high importance. Otherwise, disinfection could be insufficient. As shown in previous studies, a water-based precleaning is sufficient, and no further chemical or otherwise disinfecting agents are needed for precleaning. In this context, it should be mentioned that the tests on the MS-2 bacteriophages were conducted without precleaning. Furthermore, due to the strong bioactivity of UV radiation, despite its effectiveness against microorganisms, it can pose a danger to the user, such as the development of skin cancer [29]. Therefore, a sufficient shielding against the UV radiation is needed. In the D25, shielding is realized by a box-based design and an emergency shut-off if the device is opened during the application of the UV light. Another main issue in UV disinfection is shadowing, which might lead to insufficient disinfection [30]. Nevertheless, the results of previous studies suggest good light distribution preventing shadowing in the tested UV system [14].

## 5. Conclusions

Endoscope disinfection processes are often complex, whereas viruses and spores are the most difficult to eliminate. According to our results, it can be assumed that the UV system tested in this study has good virucidal properties. Thus, this system could offer substantial advantages over standard disinfection methods for tools such as ENT endoscopes.

## Data Availability

The data presented in this study are available on request from the corresponding author. The data are not publicly available due to trademark aspects.
